# Unlocking the role of lactate: metabolic pathways, signaling, and gene regulation in postmitotic retinal cells

**DOI:** 10.3389/fopht.2023.1296624

**Published:** 2023-11-08

**Authors:** Raju V. S. Rajala, Ammaji Rajala

**Affiliations:** ^1^ Departments of Ophthalmology, University of Oklahoma Health Sciences Center, Oklahoma City, OK, United States; ^2^ Departments of Physiology, University of Oklahoma Health Sciences Center, Oklahoma City, OK, United States; ^3^ Departments of Cell Biology, University of Oklahoma Health Sciences Center, Oklahoma City, OK, United States; ^4^ Dean McGee Eye Institute, Oklahoma City, OK, United States

**Keywords:** lactate dehydrogenase, lactate, posttranslational modifications, photoreceptor cells, glycolysis, aerobic glycolysis, Warburg effect, anabolic processes

## Abstract

The Warburg effect, which was first described a century ago, asserts that mitotic tumor cells generate higher quantities of lactate. Intriguingly, even in typical physiological circumstances, postmitotic retinal photoreceptor cells also produce elevated levels of lactate. Initially classified as metabolic waste, lactate has since gained recognition as a significant intracellular signaling mediator and extracellular ligand. This current review endeavors to provide a concise overview and discourse on the following topics: the localization of lactate-producing enzymes, the functional significance of these enzymes, the signaling functions of lactate, and its impact on the gene expression of photoreceptors in retinal cells.

## Introduction

### Historical context of lactate metabolism

Lactate was first discovered in 1780 by the Swedish chemist Karl Wilhelm Scheele, who found it in sour milk (1). Araki and Zillessen’s experiments, which disrupted the oxygen supply to muscles in birds and mammals, led to increased lactate levels, establishing a link between lactate production and tissue hypoxia (2). Lactate was traditionally viewed as a metabolic byproduct generated in hypoxic conditions characterized by low oxygen levels (1). Johann Joseph Scherer, a German physician-chemist, first identified lactate in human blood post-mortem in 1843, while Carl Folwarczny discovered it in the blood of a living patient in 1858 (2). The collaborative research by Walter Morley Fletcher (1873–1933) and Frederick Gowland Hopkins (1861–1947) at Cambridge revealed that lactate resulted from carbohydrate metabolism during muscle contraction under anaerobic conditions (3). Athletes commonly associate lactate with muscle fatigue, decreased performance, and discomfort (4). However, in 1970, George Brooks from UC Berkeley challenged this notion, demonstrating that lactate is not a waste product but rather a preferred energy source for the body (4). Interestingly, the brain and heart efficiently utilize lactate over glucose circulating in the blood (4). Lactate exhibits intracellular and intercellular movement, a phenomenon termed the “lactate shuttle,” originally proposed by Professor George Brooks at UC Berkeley (4). It is continually produced and utilized in various cells under both aerobic and anaerobic conditions (4). Furthermore, lactate is generated at sites with high glycolysis and gluconeogenesis rates and shuttles to nearby or distant locations, where it serves as a gluconeogenic precursor or an oxidation substrate (5). A century ago, Otto Henrich Warburg observed that tumor cells produce lactate in the presence of oxygen, a phenomenon now known as the Warburg effect or aerobic glycolysis (6).

**Figure 1:**
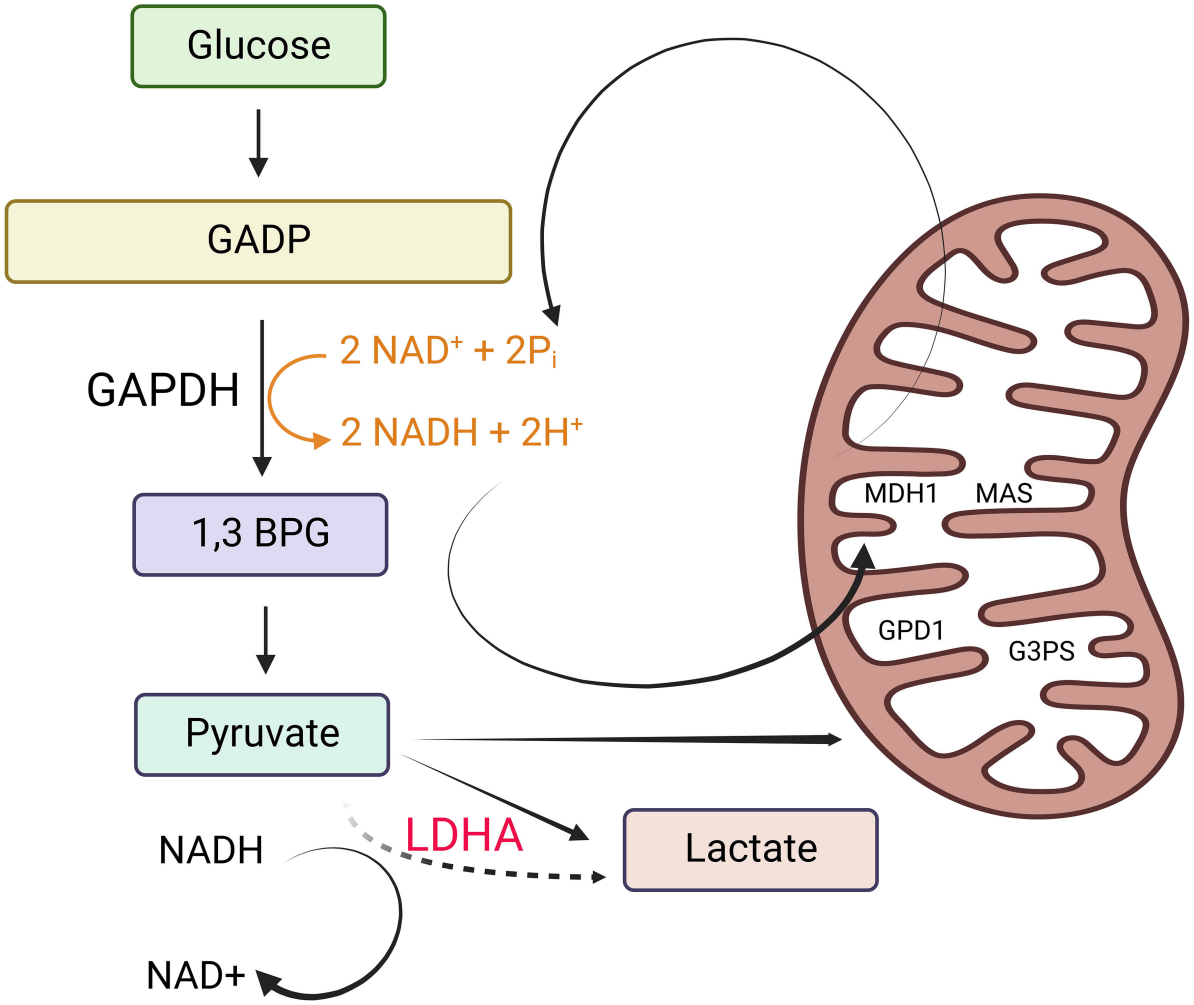
Why tumor cells generate lactate. Tumor cells produce lactate as a result of the need to maintain cytosolic NAD+ levels for the continuation of glycolysis. This is essential to support the reaction catalyzed by glyceraldehyde 3-phosphate dehydrogenase (GAPDH). In non-proliferating cells, NADH is primarily oxidized to NAD+ through malate dehydrogenase 1 (MDH1), a component of the malate-aspartate shuttle (MAS) located in the inner mitochondrial membrane. MDH1 activity in non-proliferating cells is not operating at its maximum capacity. Depending on the specific cell line and the availability of oxygen, non-proliferating cells also utilize lactate dehydrogenase (LDH) to help oxidize NADH. Additionally, they partially oxidize small amounts of NADH using glycerol 3-phosphate dehydrogenase 1 (GPD1), a component of the glycerol 3-phosphate shuttle (G3PS) in the inner mitochondrial membrane. However, in proliferating cells that exhibit the Warburg effect (characterized by high rates of glycolysis), NADH levels increase significantly, creating a greater demand for NADH oxidation. When the processes involving NAD+ regeneration via the MAS and the G3PS reach their maximum capacity due to the saturation of MDH1 and GPD1, the expression and activity of LDH increase. This heightened LDH activity facilitates the oxidation of excess NADH in the cytosol, leading to an increase in lactate production. This figure was created with BioRender.com.

The enigma within the tumor biology domain revolved around why mitotic tumor cells, instead of utilizing glucose for oxidative phosphorylation (OXPHOS), opt to produce lactate from it. Warburg’s research findings pointed to cancer cells altering their ATP production pathway from OXPHOS to aerobic glycolysis due to mitochondrial dysfunction (7, 8). Various hypotheses have emerged to elucidate the Warburg effect. These hypotheses encompass the rapid synthesis of ATP, the generation of essential building blocks for biosynthesis, acidification of the microenvironment, metabolic interactions, and cellular signaling via the regulation of reactive oxygen species and histone acetylation (8). Almost a century later, in 2022, the scientific community arrived at a consensus regarding the purpose of aerobic glycolysis (8). This study’s conclusion underscores that lactate fermentation becomes a secondary mechanism activated when the mitochondrial transporters surpass their capacity to oxidize cytosolic NADH (8) (**Figure 1**).

Nicotinamide adenine dinucleotide (NAD+) functions as a coenzyme with a crucial role in glycolysis (9). To sustain glycolysis, it is essential to maintain cytosolic NAD+ levels, which are necessary for the reaction facilitated by glyceraldehyde 3-phosphate dehydrogenase (G3PD). In non-proliferating cells, the oxidation of NADH back to NAD+ primarily occurs through malate dehydrogenase 1 (MDH1), a constituent of the malate-aspartate shuttle (MAS) located in the inner mitochondrial membrane. MDH1 activity remains unsaturated in non-proliferating cells. Depending on the specific cell line and the presence of oxygen, non-proliferating cells also employ lactate dehydrogenase (LDH) for NADH oxidation. Additionally, they utilize glycerol 3-phosphate dehydrogenase 1 (GPD1), a component of the glycerol 3-phosphate shuttle (G3PS) within the inner mitochondrial membrane, to oxidize small amounts of NADH.

In actively dividing cells displaying the Warburg effect, the elevated glycolytic rates result in increased NADH levels, intensifying the demand for NADH oxidation. When the regeneration of NAD+ by the mitochondrial electron transport chain (MAS) and the cytosolic glycerol-3-phosphate shuttle (G3PS) reaches its maximum capacity due to the saturation of MDH1 and GPD1, there is an upregulation in LDH expression and activity to facilitate the oxidation of surplus NADH in the cytoplasm. This leads to an increase in lactate production (8). Consequently, the conversion of glucose to lactate, which is a characteristic feature of cancer, arises as a secondary outcome of the saturation of MAS and G3PS, rather than being the primary metabolic driver of cell proliferation.

**Figure 2:**
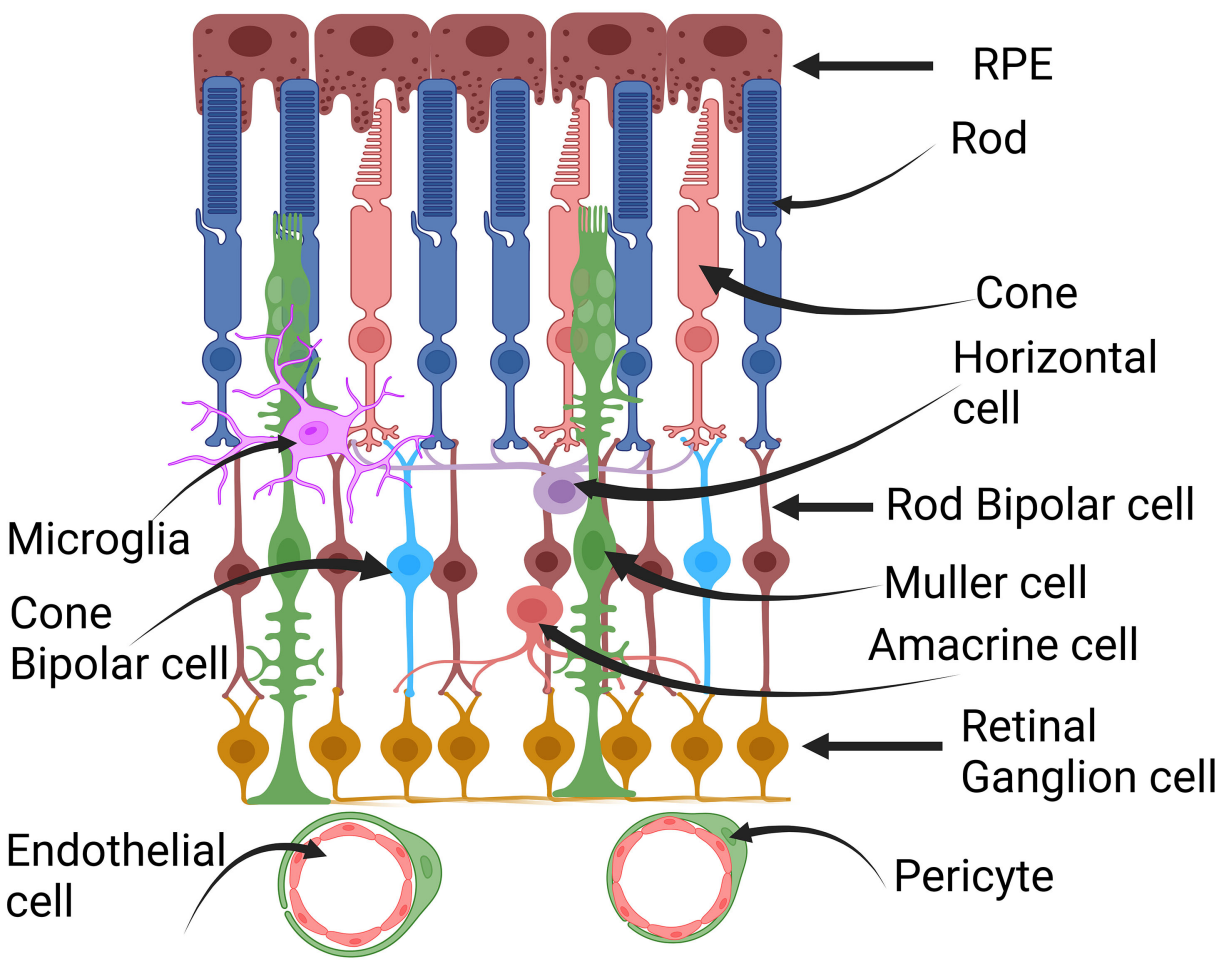
Cross-section of the retina. Retina is a complex tissue comprising various cell types. These include retinal pigment epithelial cells (RPE), rod and cone photoreceptor cells, horizontal cells, rod and cone bipolar cells, Müller cells, amacrine cells, retinal ganglion cells, microglia, pericytes, and endothelial cells. This figure was created with BioRender.com.

The retina, located at the back of the eye, is a complex tissue essential for vision. It converts light signals into electrical impulses, sending them to the brain so we can perceive visual information. The retina is made up of various cells, including retinal pigment epithelial cells (RPE), rod and cone photoreceptor cells, rod bipolar cells, cone bipolar cells, horizontal cells, amacrine cells, Müller cells, and retinal ganglion cells (10, 11) (**Figure 2**). These different cell types collaborate to capture and process visual information, allowing us to see objects clearly. Numerous comprehensive reviews exist that delve into lactate and its implications for tumor progression. However, this particular review primarily concentrates on the role of lactate in the postmitotic retina in comparison to its role in mitotic tumor cells. As observed by Warburg, postmitotic retinal cells consistently undergo glucose fermentation to lactate even in the presence of oxygen, a phenomenon known as aerobic glycolysis. Aerobic glycolysis is indispensable for postmitotic photoreceptor cells, especially as they engage in phagocytosis when exposed to light (12, 13). About 10% of photoreceptors are phagocytosed by the adjacent retinal pigment epithelium (RPE), which intricately interacts with photoreceptors (14, 15). To sustain the length of photoreceptor cells, there is a necessity to synthesize substantial amounts of lipids, RNA, and DNA. Consequently, glucose is redirected for macromolecular synthesis rather than being utilized for oxidative phosphorylation (OXPHOS). Moreover, aerobic glycolysis also supports the pentose phosphate pathway, which generates NADPH, essential for antioxidant metabolism (12). In instances of temporary nutrient scarcity, the mitochondria in photoreceptor cells provide an alternative route for NADPH production (16). The photooxidation of rhodopsin results in the formation of harmful molecules that require detoxification, with NADPH playing a crucial role in reducing oxidized glutathione to its reduced form, a major antioxidant (12). Additionally, the photolysis of rhodopsin produces substantial quantities of all-trans-retinal, necessitating NADPH for its reduction to all-trans-retinal, catalyzed by Retinol Dehydrogenase 8 (RDH8) (12). Any disruption in this process leads to the production of a group of toxic retinoids that accumulate in the RPE and ultimately culminate in RPE cell death (17, 18).

A distinctive variant of LDH, known as LDH k, is notably abundant in cultured cells that have undergone transformation due to the Kirsten murine sarcoma virus (19). Researchers have also detected increased levels of LDH k activity in extracts obtained from normal rodent retinas (19). This activity shares essential characteristics with the isozyme identified in human tumors, including a strongly cathodic electrophoretic mobility and sensitivity to inhibition by oxygen and 5’,5’-dipurinenucleoside tetraphosphates. The presence of this activity in the retina may be connected to the heightened aerobic glycolysis observed in retinas, a metabolic feature commonly observed in various tumor types (19).

The genes LDHA and LDHB encode the tetrameric enzyme lactate dehydrogenase, which facilitates the conversion between pyruvate and lactate using the co-substrate NADH/NAD+ (20). The LDHA homotetramer is responsible for converting pyruvate to lactate with NADH as a cofactor, while LDHB primarily catalyzes the conversion of lactate to pyruvate with NAD as a cofactor (21). LDHB has been demonstrated to partially substitute for LDHA in promoting glycolysis, and the simultaneous deletion of LDHA and LDHB has been shown to suppress LDH activity and lactate secretion (22). The LDHA gene is activated in response to hypoxia, whereas under hypoxic conditions, the expression of LDHB is downregulated in primary cultures of rat and chick retinas (23). In the developing chick retina, LDHB is found in regions with ample oxygen supply, where active mitochondria are located (23).

## Localization of LDHA and LDHB in the retina

LDH was identified in various ocular structures, including the corneal epithelium, corneal endothelium, ciliary muscle, non-pigmented ciliary epithelium, iris muscle, lens epithelium, lens cortex, pigment epithelium, and the neurosensory retina (24). This immunohistochemical localization emphasizes that the enzyme is predominantly distributed in ocular structures exhibiting heightened metabolic activity (24).

**Figure 3:**
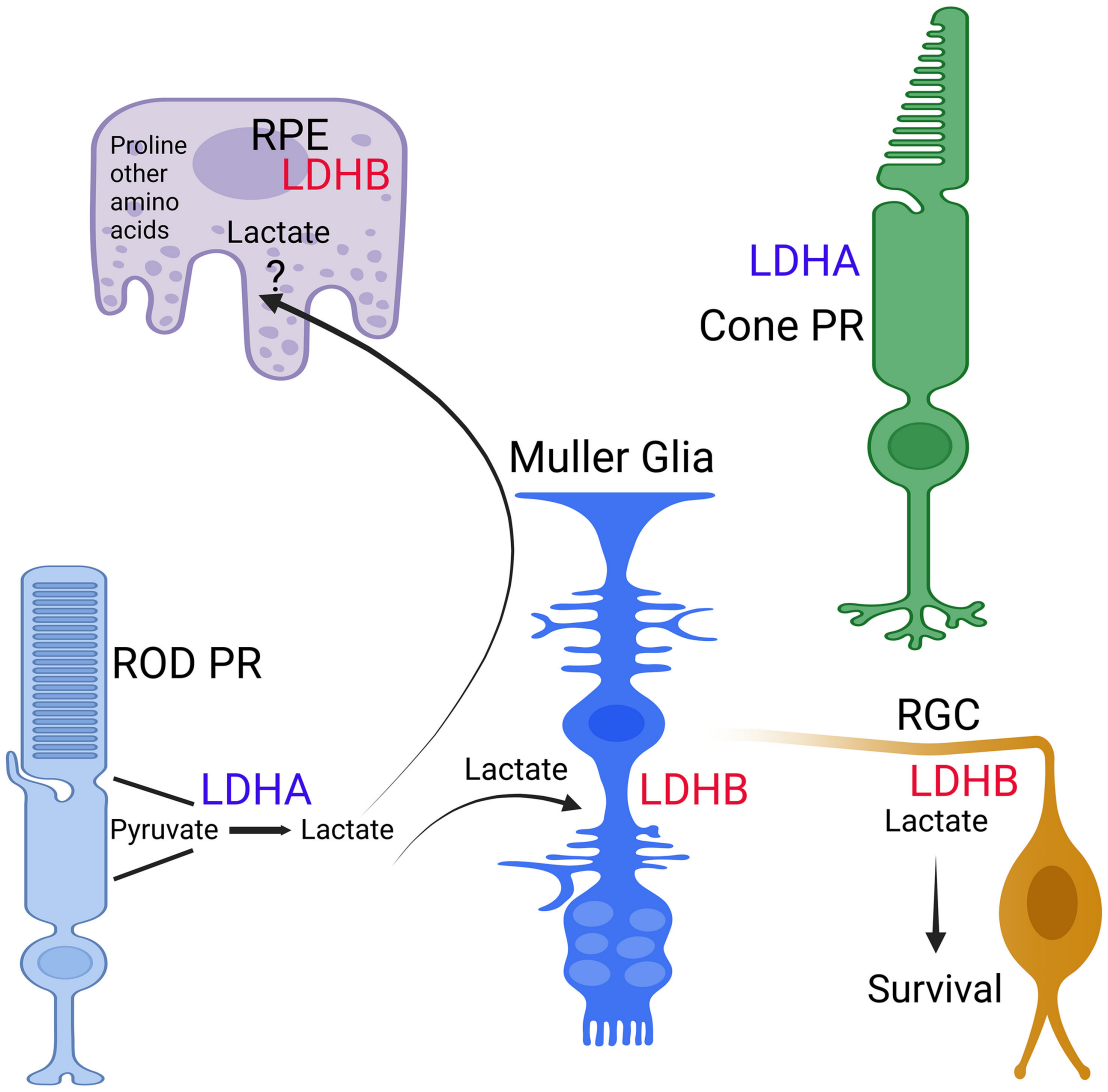
Expression of LDH isoforms in retina cells. The expression of LDHA and LDHB in RPE, Rods, Cones, Muller cells, and ganglion cells was determined through TRAP analysis (27, 28). This figure was created with BioRender.com.

In the avascular rabbit retina, LDHA expression is primarily observed within photoreceptor inner segments (25). Lactate production in photoreceptors relies on LDHA (26), indicating the presence of LDHA in photoreceptor cells. One challenge in identifying LDHA and LDHA isoforms in retinal cells has been the lack of specific antibodies or potential cross-reactivity of LDHB with LDHA (27). These limitations have been overcome with the introduction of Translating Ribosome Affinity Purification technology (27, 28). This method identifies actively translating mRNA in a cell-specific manner (29). Through this technology, it becomes evident that rod and cone photoreceptors exhibit higher levels of LDHA expression, whereas RPE, Müller cells, and retinal ganglion cells (RGC) exhibit higher levels of LDHB expression (27) (**Figure 3**). Among these, RPE demonstrates the highest levels of LDHB expression (27) (**Figure 2**). LDHA is predominantly expressed in the outer retina, while LDHB expression is localized to the inner retina (27). Developmental studies indicate that LDHA expression initiates before LDHB during development (27). This observation suggests that LDHA plays an essential role in aerobic glycolysis to support macromolecular synthesis. In tumor cells, LDHA undergoes tyrosine phosphorylation at position Y10, contributing to tumorigenesis (30). In the context of the retina, inhibition of fibroblast growth factor has been shown to reduce the phosphorylation of LDHA at Y10 (26), implying that LDHA undergoes tyrosine phosphorylation not only in mitotic cells but also in postmitotic cells.

## The fate of lactate in the retina

The retinal pigment epithelium (RPE) functions as the outer blood-retina barrier and aids in glucose transportation across the epithelium into the outer retina using GLUT1 (31). Glucose undergoes various metabolic processes in photoreceptors, including the tricarboxylic acid cycle (TCA) and oxidative phosphorylation (OXPHOS) (32). Furthermore, photoreceptors employ aerobic glycolysis to generate glycerol, essential for renewing phospholipids in their outer segments (32). This process also results in the rapid production of lactate, which the RPE transports out of the subretinal space into the choroidal circulation (32). Within the RPE, absorbed lactate is converted into pyruvate and metabolized through oxidative phosphorylation (32). Any surplus lactate within the RPE is transferred across the basolateral membrane to the choroid (32). The uptake of glucose by cone photoreceptor cells is facilitated by the rod-derived cone viability factor (RdCVF) secreted by rods and insulin signaling (32). Studies involving biochemical and nuclear magnetic resonance measurements of glucose utilization in the cone-dominant ground squirrel retina have established that lactate, rather than CO2, serves as the primary product of glucose metabolism (33). Hurley’s laboratory has demonstrated the existence of a metabolic ecosystem in the vertebrate retina (34). Glucose transported into the photoreceptors via glucose transporter 1 (GLUT1) is metabolized into lactate, which is then transported to RPE and Müller cells via monocarboxylate transporters (MCT). Within these cells, it is converted to pyruvate to power their mitochondria (35).

A metabolic interdependence between neurons and glia in the retina has been established (36). Specifically, Müller glia lacks pyruvate kinase and aspartate/glutamate carrier 1 (AGC1), which are crucial components of the malate aspartate shuttle. In contrast, photoreceptor neurons express the M2 isoform of pyruvate kinase and AGC1 (37). Consequently, Müller glia addresses their distinct metabolic needs by utilizing lactate and aspartate derived from neurons as substitutes for the missing pyruvate kinase and AGC1 (37). This metabolic ecosystem ensures efficient glucose utilization for photoreceptor survival. Furthermore, the extracellular interphotoreceptor matrix (IPM), located between photoreceptors and RPE, serves as a pathway for outer-retinal nutrition (38).

MCTs, a group of transporters, currently comprise 14 members (39). Nevertheless, only MCT1–4 have been subjected to biochemical characterization (39). Despite the shared ability of MCTs 1-4 to transport lactate, they exhibit variations in their transport mechanisms and are distributed across diverse tissues and cellular compartments (39). Their pivotal role is in facilitating lactate exchange between cells reliant on glycolysis and those reliant on oxidative metabolism within tissues and between blood-tissue barriers (40). The deletion of basigin, an accessory protein associated with monocarboxylate transporters (MCT 1, 3, and 4) responsible for lactate transport in rod cells, leads to retinal degeneration (41). Aquaporin 9 (AQP9) belongs to the aquaglyceroporin category and is recognized for its lactate transport capacity (42). Accumulating evidence supports the significance of the astrocyte-to-neuron lactate shuttle (ANLS) in neuronal energy metabolism, particularly in retinal ganglion cells (RGCs). This substantiates the role of Aqp9 in facilitating the lactate transfer between astrocytes and neurons, in conjunction with monocarboxylate transporters (42). In line with this hypothesis, the deletion of the Aqp9 gene exacerbates the death and impairment of retinal ganglion cells (RGCs) resulting from optic nerve crush (42). Recent research has revealed that these MCT transporters are not exclusive to lactate, as MCT1 is involved in succinate export in the retina (43). Notably, succinate, another metabolic intermediate, exits retinal tissue through MCT1 but does not enter the retinal pigment epithelium (RPE) via MCT1 or any other MCT, suggesting the presence of an as-yet-unidentified transporter that facilitates succinate import into the RPE (43).

## The functional roles of lactate in retinal cells

Introducing sequencing-specific LDHA-shRNA into the retina results in a reduction in the length of the outer segment (26). This discovery highlights the significance of aerobic glycolysis in a metabolic scheme that meets the biosynthetic demands of typical neuronal cell types. Deleting LDHA specifically in the retina leads to age-related visual function deterioration, followed by retinal degeneration (27). A debate in the field arose when it was proposed that lactate, released by Müller glial cells, is metabolized by photoreceptors in the mammalian retina (44, 45). This issue was deliberated in the context of the metabolic ecosystem, suggesting that lactate produced by photoreceptors is transported to Müller glia (34), where it is converted to pyruvate using LDHB to fuel their mitochondria due to the higher expression of LDHB compared to LDHA (27). Two separate studies have demonstrated that specific deletion of LDHA in Müller cells does not yield any detectable phenotypes (27, 46). Interestingly, Müller cells alter their characteristics from oxidative phosphorylation (OXPHOS) to glycolysis in culture, utilizing glucose to produce lactate (27). It is intriguing to note that *in vivo*, Müller glia do not complete glycolysis but instead utilize glucose to generate serine, which plays a crucial role in supporting photoreceptor health (46). These observations further substantiate earlier findings, suggesting that the lactate produced in photoreceptor cells is transported to Müller cells to provide fuel for the mitochondria (35). Notably, alterations in LDHA isoenzymes in the retinas of sheep are linked to progressive retinal degeneration, commonly known as Bright Blindness (47). The enzyme changes observed in the retinal dystrophy of Bright Blindness closely resemble the enzyme changes seen in both retinitis pigmentosa and sodium iodate toxicity (47).

## Altered retinal metabolism in LDHA-deficient mouse retina

**Figure 4:**
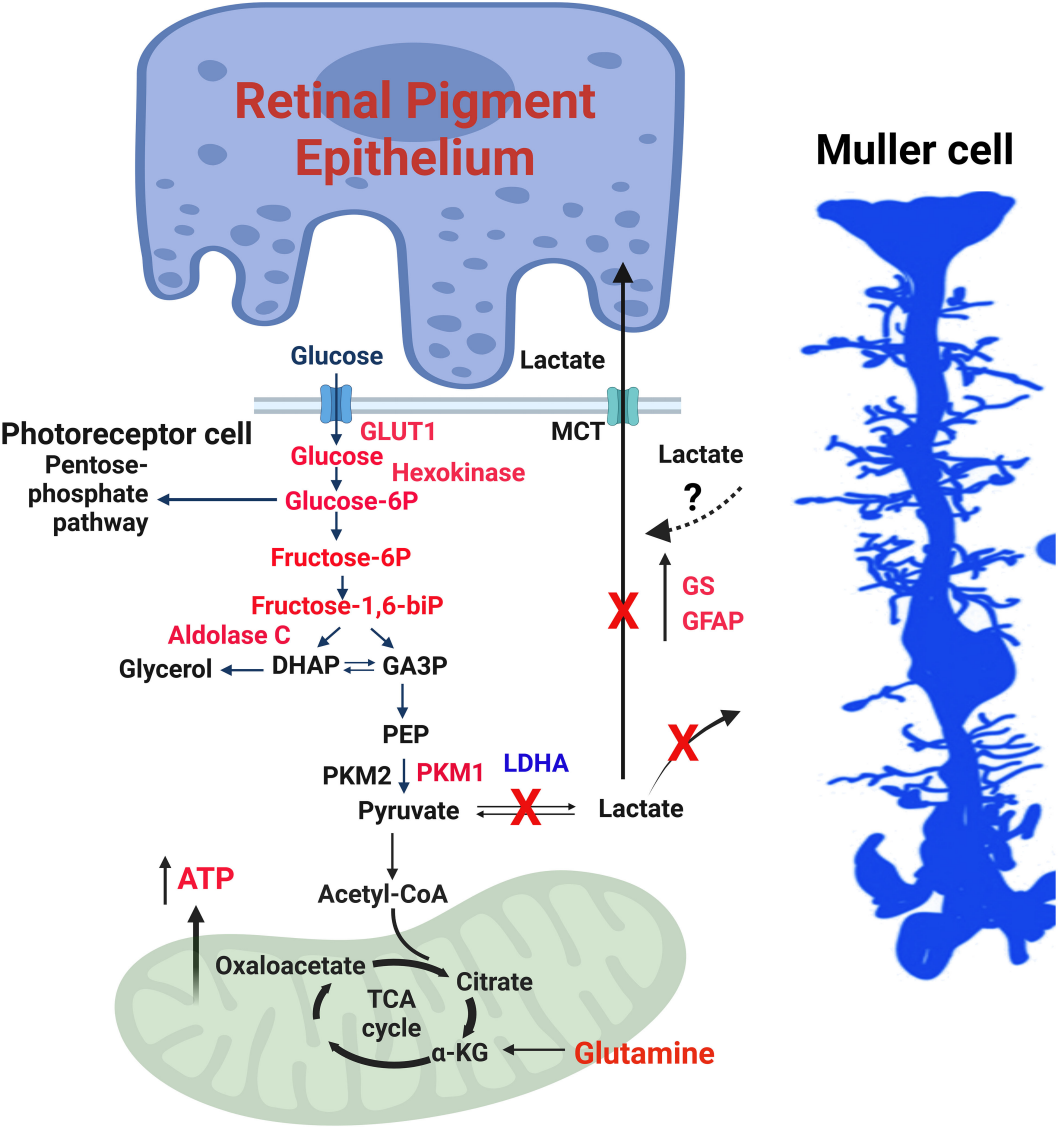
LDHA-mediated functions in the retina. The enzymes, glycolytic intermediates and the metabolites that are increased in mouse retinas lacking LDHA are shown in red. 1) Photoreceptor cells use glucose to produce lactate even when oxygen is present, a phenomenon called the “Warburg effect” or aerobic glycolysis. 2) Glucose is supplied to photoreceptor cells by RPE cells through a transporter called GLUT1. LDHA converts pyruvate, a product of glycolysis, into lactate. 3) The lactate generated by photoreceptor cells is transported to RPE and Müller cells via monocarboxylate transporters (MCT). In these cells, lactate is converted back to pyruvate, which fuels mitochondria for oxidative phosphorylation. 4) In photoreceptor cells, the enzyme PKM2 converts phosphoenolpyruvate (PEP) to pyruvate. Phosphorylation of PKM2 reduces its affinity for PEP, leading to an accumulation of PEP and activation of the pentose phosphate pathway, similar to what happens in cancer cells. 5) Fructose 1,6-bisphosphate (FBP) activates PKM2, and aldolase C breaks down FBP into dihydroxy acetone phosphate (DHAP) and glyceraldehyde 3-phosphate (GA3P). 6) The absence of LDHA in the retina results in increased levels of glycolytic intermediates and enzymes, along with increased expression of PKM1 and reduced PKM2 expression, favoring oxidative phosphorylation. 7) Müller cells exhibit increased expression of glutamine synthetase (GS) and glial fibrillary acidic protein (GFAP), along with higher glutamine levels. 8) Loss of LDHA leads to decreased visual function and disrupts the dorsal-ventral pattern of the cone-opsin gradient. 9) Deleting LDHA in Müller cells doesn’t impact visual function, indicating that the lactate produced in photoreceptors is transported to Müller cells. This figure was created with BioRender.com. GLUT1, Glucose transporter 1; MCT, Monocarboxylate transporter; PKM2, Pyruvate kinase M2 isoform; PKM1, Pyruvate kinase M1 isoform; DHAP, Dihydroxy acetone phosphate; GA3P, Glyceraldehyde 3-phosphate; PEP, Phosphoenolpyruvate; LDHA, Lactate dehydrogenase A; GS, Glutamine synthetase; GFAP, Glial fibrillary acidic protein; ATP, Adenosine triphosphate; FBP, Fructose 1,6-bisphosphate.

The absence of LDHA leads to the upregulation of Müller cell markers, such as glutamine synthetase (GS) and glial fibrillary acidic protein (GFAP) (27) (**Figure 4**). LDHA knockout (KO) mice at younger age do not exhibit significant changes in the expression of photoreceptor-specific proteins, including rhodopsin, rod arrestin, PDE6β, M-opsin, and cone arrestin (27). Pyruvate kinase M2 (PKM2) is predominantly expressed in photoreceptor cells, whereas PKM1 is primarily localized to the inner retina, particularly in the inner plexiform and ganglion cell layers (48). PKM2 undergoes phosphorylation at tyrosine 105, reducing its affinity for phosphoenolpyruvate (PEP) and activating the pentose phosphate pathway in tumor cells (49). While a tissue may potentially contain multiple pyruvate kinase isoforms, individual cells typically express only one isoform at significant levels (50). In LDHA-deficient retinas, there is a decrease in PKM2 expression and PKM2 phosphorylation, leading to the expression of both PKM1 and PKM2 in photoreceptors (27) (**Figure 3**). LDHA KO retinas also display elevated levels of fructose 1,6-bisphosphate (FBP) (27), serving as an allosteric activator of PKM2 with a higher affinity for PEP, thus generating pyruvate (51, 52). FBP is enzymatically cleaved by fructose-bisphosphate aldolase (aldolase), yielding 3-carbon sugars, glyceraldehyde 3-phosphate (G3P), and dihydroxyacetone phosphate (DHAP) (53). Both DHAP and G3P serve as precursors for glycerol synthesis, forming the essential backbone of lipids (27).

In LDHA-deficient retinas, there is an elevation in protein levels of hexokinase 1, aldolase C, and Glut1, accompanied by increased activities of hexokinase, pyruvate kinase, and aldolase (27) (**Figure 3**). Remarkably, LDHA-deficient retinas demonstrate reduced lactate/pyruvate and succinate/fumarate ratios, indicating a decreased reduction potential within the retina, likely concentrated in the rods, compared to the typical NADH/NAD ratio. Under these conditions, steady-state ATP levels increase (27). This could potentially occur if rod cells possessed a greater number of mitochondria or if the mitochondria were more actively involved in the oxidation of NADH to NAD than usual. Supporting this hypothesis, LDHA knockout retinas exhibit increased protein levels of pyruvate dehydrogenase (27).

The steady-state metabolite profile in LDHA KO retinas reveals elevated levels of glucose, glucose 6-phosphate, fructose, and fructose-6-phosphate, alongside decreased levels of lactate and proline (27). Interaction analysis of steady-state metabolites suggests potential changes centered around ATP (27). The increased glucose levels in LDHA KO retinas may be attributed to decreased glucose utilization.

## Lactate-dependent transcriptional regulation

**Figure 5:**
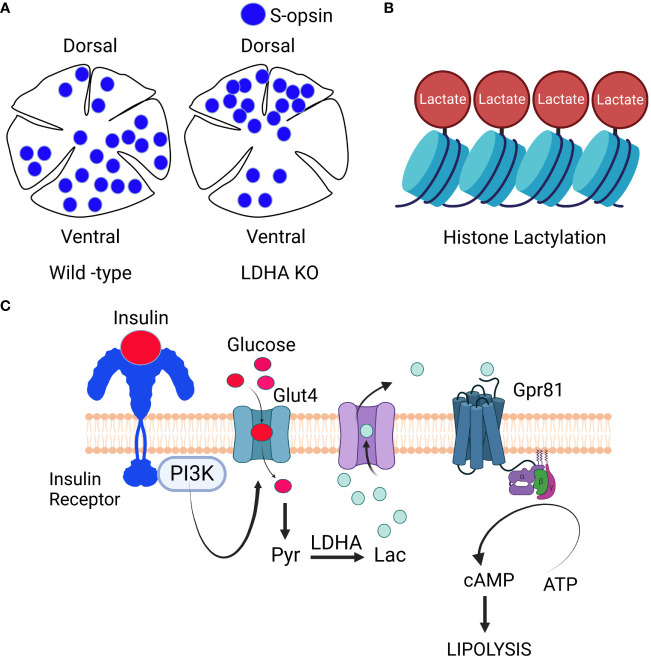
Signaling through Lactate: LDHA regulates cone-opsin gradient: LDHA plays a role in regulating the dorso-ventral patterning of the S-opsin gradient **(A)**. Lactate-Mediated Histone Modification: Lactate is also involved in histone modification, a process known as histone lacylation **(B)**. Signaling role of lactate: When growth factors activate phosphoinositide 3-kinase (PI3K), it triggers the movement of glucose transporters to the cell’s plasma membrane. Glucose then enters the cell and undergoes metabolism, resulting in the generation of lactate. This lactate exits the cell and binds to the orphan receptor Gpr81. This binding inhibits the activity of membrane-bound adenylate cyclase, ultimately reducing the synthesis of cAMP **(C)**. The insulin/insulin receptor/PI3K/Glut4/lactate/Gpr81 pathway, known for its role in inhibiting lipolysis in adipocytes (54), is yet to be conclusively established in the retina. Nonetheless, research has identified Gpr81 expression in Müller cells and retinal ganglion cells (55), along with the presence of insulin-responsive glucose transporter GLUT4 in these cell layers (56)). Intriguingly, the loss of insulin receptors in Müller glia has been associated with retinal degeneration (46), and insulin signaling is being explored as a potential therapy for glaucomatous neurodegeneration (57). It is worth noting that fatty acid β-oxidation enzymes are predominantly located in the mitochondria of Müller cells (58). These findings raise the intriguing possibility that the lactate-mediated suppression of lipolysis observed in adipocytes might also occur in Müller glia and retinal ganglion cells within the retina. This figure was created with BioRender.com.

Recent investigations have established that LDHA plays a role in governing the dorsal-ventral pattern of the cone-opsin gradient (27) (**Figure 5**). The ventral part of the retina predominantly expresses S-opsin-positive cones when compared to the dorsal portion (59). M-opsin-positive cones are evenly distributed in both regions but exhibit slightly greater dispersion in the dorsal part of the retina. The presence of LDHA knockout results in a higher distribution of S-opsin-positive cones in the dorsal region and reduced expression in the ventral region (27). Importantly, the absence of LDHA does not impact the distribution of M-opsin in the retina (27). LDHA primarily converts lactate to pyruvate in the cytosol, but in the nucleus, it regulates cell cycle progression by enhancing SIRT1 activity through NAD+ supplementation (60–62). Altering metabolism by specifically targeting sirtuin 6 has been shown to reduce the severity of retinal degeneration (63). LDHA also interacts with single-stranded DNA, assisting in DNA replication by recruiting DNA polymerases (60–62). Recent findings indicate that lactate-dependent transcriptional regulation plays a critical role in mammalian eye development (64). The absence of Glut1 and LDHA from retinal progenitor cells leads to arrested eye development (65). These studies unveil an unexpected and unidentified non-metabolic role of lactate as an indispensable signaling molecule in mammalian eye development (64).

In recent research, lactate has been found to have a non-metabolic function in addition to its well-known metabolic roles. Lactate now emerges as an epigenetic modifier by lactylating histone lysine residues, directly initiating gene transcription from chromatin (66) (**Figure 4**). Scientists have identified 28 specific lactylation sites on core histones in human and mouse cells (66). Conditions such as hypoxia and bacterial challenges stimulate lactate production via glycolysis, which then serves as a precursor for histone lactylation (66). This discovery of histone lactylation provides a fresh perspective on our understanding of lactate’s multifaceted functions and its involvement in various pathophysiological conditions, including retinal degeneration.

## Signaling roles of lactate

Emerging evidence suggests that substances previously regarded solely as fuel, waste, or metabolic intermediates now play a crucial role in orchestrating physiological processes through cellular signaling. In addition to its role as a metabolic fuel, lactate also serves as a ligand for G-protein-coupled receptor 81 (GPR81) and inhibits lipolysis by suppressing cAMP formation via adenylate cyclase inhibition in adipocytes (54, 67) (**Figure 4**). Elevated cAMP levels are known to initiate signals within rod cells that ultimately lead to caspase 3 activation and apoptotic cell death (65, 68).

The insulin/insulin receptor/PI3K/Glut4/lactate/Gpr81 pathway was initially identified in adipocytes, where it plays a role in blocking lipolysis (54) (**Figure 5**). However, this pathway has not yet been confirmed to exist in the retina. Nevertheless, research has shown that Gpr81 is expressed in Müller cells and retinal ganglion cells (55), and the insulin-responsive glucose transporter GLUT4 is also found in these cell layers (56). Interestingly, the loss of insulin receptors in Müller glia has been linked to retinal degeneration (46), while insulin signaling has been identified as a potential target for treating glaucomatous neurodegeneration (57). Notably, fatty acid β-oxidation enzymes are mainly located in the mitochondria of Müller cells (58). These findings strongly suggest that the lactate-mediated inhibition of lipolysis seen in adipocytes might also occur in Müller glia and retinal ganglion cells.

Lactate and its receptor GPR81 are indispensable for the development of the visual nervous system (69). Similarly, GPR43 sensing of acetate triggers an alert in neutrophils and provides protection against severe sepsis (70). During infancy, the interaction between propionate and GPR41 offers protection against the later onset of bronchial asthma (71). Recent research has revealed that the α-ketoglutarate-GPR99 axis regulates the pathological phenotype of cardiac hypertrophy in mice (72). The activation of the receptor GPR109A by β-Hydroxybutyrate plays a role in regulating fasting-induced changes in the plasticity of the mouse adrenal medulla (73). The succinate receptor GPR91 is expressed in various tissues and cell types (74), and GPR91 signaling modulates pathological processes in chronic illnesses (74). Human DNA sequence variants in the Gpr91 gene are associated with atrophic age-related macular degeneration (AMD) (75). Mice lacking GPR91 exhibit retinal degeneration of the outer retina, accumulation of oxidized low-density lipoproteins in the subretinal space, Bruch’s membrane thickening, and elevated levels of microglia in the subretinal space, mimicking the AMD phenotype (75). The bile acid/Takeda G protein-coupled receptor 5 (TGR5) signaling regulates fatty liver disease (76). Long-chain fatty acids bind to free fatty acid receptor 4 (FFA4), also known as GPR120, and regulate glucose homeostasis (77). In the retina, retinal lipid and glucose metabolism influence the process of angiogenesis through the lipid sensor free fatty acid receptor 1 (FFAR1) (78). In the gut, these GPRs provide anti-inflammatory signals (79), and a similar role for these receptors in the retina should not be overlooked. Future studies should aim to elucidate how these metabolites regulate cellular signaling and identify the downstream effector molecules that govern physiological processes.

## Therapeutic advantages of lactate

Lactate plays a pivotal role in enhancing the survival of retinal ganglion cells (RGCs) during periods of glucose deprivation (80). Research has revealed the expression of the lactate receptor GPR81 in Müller cells and retinal ganglion cells (55). Activation of GPR81 shields RGCs from glutamate-induced retinal degeneration and prevents visual impairment (81). GPR81 activation results in a decrease in cAMP levels in both neural and adipose tissues (55). Furthermore, GPR81 exerts its influence through various downstream mechanisms to regulate functions such as excitability, metabolism, and inflammation (55). In the retina, much like in the brain, lactate traverses cell membranes via MCT transporters and diffuses in the extracellular space (55). This lays the groundwork for the role of lactate as a transmitter of metabolic signals. We anticipate that targeting lactate receptors and transporters could open promising avenues for innovative therapeutic interventions. Such strategies could help safeguard neurons and prevent or ameliorate conditions like glaucoma and other retinal diseases. Other molecules, such as modified visual arrestin, increase lactate production and decelerate retinal degeneration in the P23H rhodopsin model of *retinitis pigmentosa* (82). Lactate shields the retina from degeneration induced by oxidative stress in age-related macular degeneration (AMD) by stimulating autophagy activation (83). Within the inner retina, lactate assumes a vital role as an essential energy source (84). It offers protection against both glutamate-induced excitotoxicity and ischemic damage (84). Additionally, lactate functions as a signaling molecule that effectively regulates various cellular functions (84). The administration of AAV-Thioredoxin interacting protein (Txnip) treatment in mouse models of retinitis pigmentosa resulted in prolonged cone survival and improved vision (85). The efficacy of Txnip in rescuing cone degeneration is linked to LDHB and correlates with the presence of a greater number of functional mitochondria (85).

A recent study has shown that the degenerating rd1 mouse retina releases significantly higher levels of lactate compared to the retina of wild-type mice. This suggests that a deteriorating retina may have an even greater metabolic rate and energy demand compared to a healthy retina (86). Ciliary neurotrophic factor (CNTF)-mediated protection of neurons involves enhanced glycolytic and anabolic processes in the deteriorating retinas of mice (87). Moreover, both LDHA transcripts and the active form of LDHA experience an upsurge under the influence of CNTF (86). It’s worth noting that this enzymatic conversion of lactate to pyruvate also generates NADH, which serves as a contributor to cellular metabolism and an electron donor in oxidative phosphorylation. Significantly, inhibiting LDH enzymatic activity led to a notable reduction in ATP production and α-ketoglutarate levels (87). This underscores the importance of LDH-catalyzed pyruvate generation for both CNTF-driven energy production and the mitochondrial citric acid cycle (TCA cycle) (87). Earlier studies have demonstrated that Metformin prevents the degeneration of RPE and photoreceptors by activating adenosine monophosphate-activated protein kinase (AMPK) signaling (88). Several studies have shown that metformin induces lactate accumulation (89, 90).

## Lactate as a biomarker for retinal diseases

Genome-wide association studies (GWAS) have pinpointed the lactate transporter SLC16A8 gene as the sole gene associated with AMD susceptibility, directly implicating photoreceptor dysfunction (88, 89). Investigating whether there is a correlation between vitreous lactate concentration and the number of occurrences of the SLC16A8 risk allele (rs8135665T) in AMD patients could potentially establish vitreous lactate as an AMD biomarker, distinct from the influence of the SLC16A8 risk allele. LDHA activity in the aqueous humor is commonly employed to differentiate retinoblastoma from other ophthalmic diseases, as it exhibits higher activity (91). Notable shifts in the redox state occur within both the retina and brain during the onset of diabetes in rats. Diabetic retinopathy is believed to originate from prolonged alterations in the metabolic processes of the retina. Elevated blood sugar levels lead to higher concentrations of glucose within cells, causing changes in how glucose is metabolized and resulting in an increase in the lactate-to-pyruvate ratio (92). The diabetic rat retina displayed elevated lactate levels, leading to a gradual reduction in the NAD+/NADH ratio. This observation suggests that disrupted glucose metabolism serves as an initial stage in the development of retinopathy (92). The use of Proton Magnetic Resonance Spectroscopy on vitreous samples from diabetic patients identified glutamate and lactate as the primary metabolites (93). These findings contribute to our understanding of the metabolic molecules involved in the pathogenesis of diabetic retinopathy. Vitreous lactate levels are commonly employed as a biomarker for diagnosing infections, especially in cases of infectious culture-negative endophthalmitis (94).

The retinal pigment epithelium (RPE) receives lactate from photoreceptors, which is utilized for oxidative phosphorylation (OXPHOS). Generally, photoreceptors rely on glycolysis, while RPE favors OXPHOS (34). Recent studies have reported impaired glycolysis in AMD patients, characterized by a high lactate/pyruvate ratio (95). A high lactate/pyruvate ratio is a well-established marker of mitochondrial impairment and indicates poor oxidative function in AMD (96). Aging RPE shifts from OXPHOS to glycolysis (97). Interestingly, hypoxic RPE cells undergo radical alterations in glucose and lipid metabolism, affecting nutrient availability for the sensory retina and promoting progressive photoreceptor degeneration (98). Clinical studies have demonstrated a connection between elevated hyperhomocysteinemia (HHcy) and AMD, which triggers the activation of several glycolytic enzymes (95). HHcy contributes to AMD by inducing a metabolic shift in mitochondria, where cells predominantly generate energy through glycolysis, leading to increased lactate production, cellular acidity, activation of angiogenesis, RPE barrier dysfunction, and CNV (95). Moreover, the presence of anti-retinal antibodies targeting glycolytic enzymes in AMD patients points to an anabolic dysregulation in AMD pathogenesis (99). Remarkably, RPE not only utilizes lactate to support its mitochondrial metabolism but also demonstrates a preference for proline utilization (100). Mutations in genes related to proline metabolism are associated with retinal degenerative diseases (100). Additionally, proline supplementation has been shown to improve the alleviation of RPE-induced vision loss (100, 101). Further research is needed to understand the underlying reasons and mechanisms behind the detrimental effects of elevated lactate levels on the RPE.

## Summary

Recent findings emphasize that lactate is not merely a metabolic byproduct, contrary to initial assumptions; it serves both metabolic and non-metabolic functions. Within the retina, LDHA is predominantly found in the outer retina, while LDHA is chiefly expressed in the inner retina. Lactate serves as the preferred energy source in Müller cells, RGCs, and RPE cells, while also acting as a ligand to activate GPR81 and enhance RGC survival. Furthermore, lactate contributes to the regulation of the dorsal-ventral gradient of cone-opsin patterning. Although the realm of histone lactylation is emerging, its exploration within the context of the retina remains an open area of investigation.

## Author contributions

RR: Conceptualization, Funding acquisition, Resources, Visualization, Writing – original draft, Writing – review & editing. AR: Resources, Visualization, Writing – review & editing.

## References

[1] Ferguson BS Rogatzki MJ Goodwin ML Kane DA Rightmire Z Gladden LB. Lactate metabolism: historical context, prior misinterpretations, and current understanding. Eur J Appl Physiol (2018) 118:691–728. doi: 10.1007/s00421-017-3795-6 29322250

[2] Kompanje EJ Jansen TC van der Hoven B Bakker J. The first demonstration of lactic acid in human blood in shock by Johann Joseph Scherer, (1814-1869) in January 1843. Intensive Care Med (2007) 33:1967–71. doi: 10.1007/s00134-007-0788-7 PMC2040486 17661014

[3] Fletcher WM. Lactic acid in amphibian muscle. J Physiol (1907) 35:247–309. doi: 10.1113/jphysiol.1907.sp001194 16992858 PMC1465827

[4] Brooks GA. The science and translation of lactate shuttle theory. Cell Metab (2018) 27:757–85. doi: 10.1016/j.cmet.2018.03.008 29617642

[5] Gladden LB. Lactate metabolism: a new paradigm for the third millennium. J Physiol (2004) 558:5–30. doi: 10.1113/jphysiol.2003.058701 15131240 PMC1664920

[6] Spencer NY Stanton RC. The Warburg effect, lactate, and nearly a century of trying to cure cancer. Semin Nephrol (2019) 39:380–93. doi: 10.1016/j.semnephrol.2019.04.007 31300093

[7] Zheng J. Energy metabolism of cancer: Glycolysis versus oxidative phosphorylation (Review). Oncol Lett (2012) 4:1151–7. doi: 10.3892/ol.2012.928 PMC3506713 23226794

[8] Wang Y Stancliffe E Fowle-Grider R Wang R Wang C Schwaiger-Haber M. Saturation of the mitochondrial NADH shuttles drives aerobic glycolysis in proliferating cells. Mol Cell (2022) 82:3270–3283.e3279. doi: 10.1016/j.molcel.2022.07.007 35973426 PMC10134440

[9] Amjad S Nisar S Bhat AA Shah AR Frenneaux MP Fakhro K. Role of NAD(+) in regulating cellular and metabolic signaling pathways. Mol Metab (2021) 49:101195. doi: 10.1016/j.molmet.2021.101195 33609766 PMC7973386

[10] Hoon M Okawa H Della Santina L Wang RO. Functional architecture of the retina: development and disease. Prog Retin Eye Res (2014) 42:44–84. doi: 10.1016/j.preteyeres.2014.06.003 24984227 PMC4134977

[11] Soucy E Wang Y Nirenberg S Nathans J Meister M. A novel signaling pathway from rod photoreceptors to ganglion cells in mammalian retina. Neuron (1998) 21:481–93. doi: 10.1016/S0896-6273(00)80560-7 9768836

[12] Punzo C Xiong W Cepko CL. Loss of daylight vision in retinal degeneration: are oxidative stress and metabolic dysregulation to blame? J Biol Chem (2012) 287:1642–8. doi: 10.1074/jbc.R111.304428 PMC3265845 22074929

[13] Rajala RVS. Aerobic glycolysis in the retina: functional roles of pyruvate kinase isoforms. Front Cell Dev Biol (2020) 8:266. doi: 10.3389/fcell.2020.00266 32426353 PMC7203425

[14] Lavail MM. Rod outer segment disk shedding in rat retina: relationship to cyclic lighting. Science (1976) 194:1071–4. doi: 10.1126/science.982063 982063

[15] Bok D. Retinal photoreceptor-pigment epithelium interactions. Friedenwald lecture. Invest Ophthalmol Vis Sci (1985) 26:1659–94. 2933359

[16] Adler L Chen C Koutalos Y. Mitochondria contribute to NADPH generation in mouse rod photoreceptors. J Biol Chem (2014) 289:1519–28. doi: 10.1074/jbc.M113.511295 PMC3894333 24297174

[17] Zhou J Kim SR Westlund BS Sparrow JR. Complement activation by bisretinoid constituents of RPE lipofuscin. Invest Ophthalmol Vis Sci (2009) 50:1392–9. doi: 10.1167/iovs.08-2868 PMC2669319 19029031

[18] Ueda K Zhao J Kim HJ Sparrow JR. Photodegradation of retinal bisretinoids in mouse models and implications for macular degeneration. Proc Natl Acad Sci USA (2016) 113:6904–9. doi: 10.1073/pnas.1524774113 PMC4922174 27274068

[19] Saavedra RA Anderson GR. A cancer-associated lactate dehydrogenase is expressed in normal retina. Science (1983) 221:291–2. doi: 10.1126/science.6857286 6857286

[20] Doherty JR Cleveland JL. Targeting lactate metabolism for cancer therapeutics. J Clin Invest (2013) 123:3685–92. doi: 10.1172/JCI69741 PMC3754272 23999443

[21] Deng H Gao Y Trappetti V Hertig D Karatkevich D Losmanova T. Targeting lactate dehydrogenase B-dependent mitochondrial metabolism affects tumor initiating cells and inhibits tumorigenesis of non-small cell lung cancer by inducing mtDNA damage. Cell Mol Life Sci (2022) 79:445. doi: 10.1007/s00018-022-04453-5 35877003 PMC9314287

[22] Ždralević M Brand A Di Ianni L Dettmer K Reinders J Singer K. Double genetic disruption of lactate dehydrogenases A and B is required to ablate the "Warburg effect" restricting tumor growth to oxidative metabolism. J Biol Chem (2018) 293:15947–61. doi: 10.1074/jbc.RA118.004180 PMC6187639 30158244

[23] Buono RJ Lang RK. Hypoxic repression of lactate dehydrogenase-B in retina. Exp Eye Res (1999) 69:685–93. doi: 10.1006/exer.1999.0745 10620398

[24] Leung RJ Rao NA. Lactate dehydrogenase in retinoblastoma: an immunohistochemical study of twelve eyes. Jpn J Ophthalmol (1986) 30:288–97. 3784141

[25] Casson RJ Wood JP Han G Kittipassorn T Peet DJ Chidlow G. M-type pyruvate kinase isoforms and lactate dehydrogenase A in the mammalian retina: metabolic implications. Invest Ophthalmol Vis Sci (2016) 57:66–80. doi: 10.1167/iovs.15-17962 26780311

[26] Chinchore Y Begaj T Wu D Drokhlyansky E Cepko CL. Glycolytic reliance promotes anabolism in photoreceptors. Elife (2017) 6:e25946. doi: 10.7554/eLife.25946 28598329 PMC5499945

[27] Rajala A Bhat MA Teel K Gopinadhan Nair GK Purcell L Rajala RVS. The function of lactate dehydrogenase A in retinal neurons: implications to retinal degenerative diseases. PNAS Nexus (2023) 2:pgad038. doi: 10.1093/pnasnexus/pgad038 36896135 PMC9991461

[28] Rajala A Rajala R Gopinadhan Nair GK Rajala RVS. Atlas of phosphoinositide signatures in the retina identifies heterogeneity between cell types. PNAS Nexus (2023) 2:pgad063. doi: 10.1093/pnasnexus/pgad063 37007713 PMC10062291

[29] Sanz E Yang L Su T Morris DR Mcknight GS Amieux PS. Cell-type-specific isolation of ribosome-associated mRNA from complex tissues. Proc Natl Acad Sci USA (2009) 106:13939–44. doi: 10.1073/pnas.0907143106 PMC2728999 19666516

[30] Jin L Chun J Pan C Alesi GN Li D Magliocca KR. Phosphorylation-mediated activation of LDHA promotes cancer cell invasion and tumour metastasis. Oncogene (2017) 36:3797–806. doi: 10.1038/onc.2017.6 PMC5501759 28218905

[31] Hsu SC Molday RS. Glycolytic enzymes and a GLUT-1 glucose transporter in the outer segments of rod and cone photoreceptor cells. J Biol Chem (1991) 266:21745–52. doi: 10.1016/S0021-9258(18)54699-8 1939198

[32] Léveillard T Philp NJ Sennlaub F. Is retinal metabolic dysfunction at the center of the pathogenesis of age-related macular degeneration? Int J Mol Sci (2019) 20:762. doi: 10.3390/ijms20030762 30754662 PMC6387069

[33] Winkler BS Starnes CA Twardy BS Brault D Taylor RC. Nuclear magnetic resonance and biochemical measurements of glucose utilization in the cone-dominant ground squirrel retina. Invest Ophthalmol Vis Sci (2008) 49:4613–9. doi: 10.1167/iovs.08-2004 18566456

[34] Kanow MA Giarmarco MM Jankowski CS Tsantilas K Engel AL Du J. Biochemical adaptations of the retina and retinal pigment epithelium support a metabolic ecosystem in the vertebrate eye. Elife (2017) 6:e28899. doi: 10.7554/eLife.28899 28901286 PMC5617631

[35] Chertov AO Holzhausen L Kuok IT Couron D Parker E Linton JD. Roles of glucose in photoreceptor survival. J Biol Chem (2011) 286:34700–11. doi: 10.1074/jbc.M111.279752 PMC3186402 21840997

[36] Hurley JB Lindsay KJ Du J. Glucose, lactate, and shuttling of metabolites in vertebrate retinas. J Neurosci Res (2015) 93:1079–92. doi: 10.1002/jnr.23583 PMC4720126 25801286

[37] Lindsay KJ Du J Sloat SR Contreras L Linton JD Turner SJ. Pyruvate kinase and aspartate-glutamate carrier distributions reveal key metabolic links between neurons and glia in retina. Proc Natl Acad Sci USA (2014) 111:15579–84. doi: 10.1073/pnas.1412441111 PMC4217417 25313047

[38] Adler AJ Southwick RE. Distribution of glucose and lactate in the interphotoreceptor matrix. Ophthalmic Res (1992) 24:243–52. doi: 10.1159/000267174 1436983

[39] Felmlee MA Jones RS Rodriguez-Cruz V Follman KE Morris ME. Monocarboxylate transporters (SLC16): function, regulation, and role in health and disease. Pharmacol Rev (2020) 72:466–85. doi: 10.1124/pr.119.018762 PMC7062045 32144120

[40] Adijanto J Philp NJ. The SLC16A family of monocarboxylate transporters (MCTs)–physiology and function in cellular metabolism, pH homeostasis, and fluid transport. Curr Top Membr (2012) 70:275–311. doi: 10.1016/B978-0-12-394316-3.00009-0 23177990

[41] Han JYS Kinoshita J Bisetto S Bell BA Nowak RA Peachey NS. Role of monocarboxylate transporters in regulating metabolic homeostasis in the outer retina: Insight gained from cell-specific Bsg deletion. FASEB J (2020) 34:5401–19. doi: 10.1096/fj.201902961R PMC7849204 32112484

[42] Mori S Kurimoto T Miki A Maeda H Kusuhara S Nakamura M. Aqp9 gene deletion enhances retinal ganglion cell (RGC) death and dysfunction induced by optic nerve crush: evidence that aquaporin 9 acts as an astrocyte-to-neuron lactate shuttle in concert with monocarboxylate transporters to support RGC function and survival. Mol Neurobiol (2020) 57:4530–48. doi: 10.1007/s12035-020-02030-0 PMC7515957 32748371

[43] Bisbach CM Hass DT Thomas ED Cherry TJ Hurley JB. Monocarboxylate transporter 1 (MCT1) mediates succinate export in the retina. Invest Ophthalmol Vis Sci (2022) 63:1. doi: 10.1167/iovs.63.4.1 PMC8976921 35363247

[44] Poitry-Yamate CL Poitry S Tsacopoulos M. Lactate released by Müller glial cells is metabolized by photoreceptors from mammalian retina. J Neurosci (1995) 15:5179–91. doi: 10.1523/JNEUROSCI.15-07-05179.1995 PMC6577914 7623144

[45] Tsacopoulos M Poitry-Yamate CL Macleish PR Poitry S. Trafficking of molecules and metabolic signals in the retina. Prog Retin Eye Res (1998) 17:429–42. doi: 10.1016/S1350-9462(98)00010-X 9695799

[46] Shen W Lee SR Mathai AE Zhang R Du J Yam MX. Effect of selectively knocking down key metabolic genes in Müller glia on photoreceptor health. Glia (2021) 69:1966–86. doi: 10.1002/glia.24005 33835598

[47] Sweasey D Patterson DS Terlecki S. Lactate dehydrogenase (LDH) isoenzymes in the retina of the sheep and changes associated with progressive retinal degeneration (Bright Blindness). Exp Eye Res (1971) 12:60–9. doi: 10.1016/0014-4835(71)90129-1 5120353

[48] Rajala RV Rajala A Kooker C Wang Y Anderson RE. The Warburg effect mediator pyruvate kinase M2 expression and regulation in the retina. Sci Rep (2016) 6:37727. doi: 10.1038/srep37727 27883057 PMC5121888

[49] Iqbal MA Siddiqui FA Gupta V Chattopadhyay S Gopinath P Kumar B. Insulin enhances metabolic capacities of cancer cells by dual regulation of glycolytic enzyme pyruvate kinase M2. Mol Cancer (2013) 12:72. doi: 10.1186/1476-4598-12-72 23837608 PMC3710280

[50] Israelsen WJ Vander Heiden MG. Pyruvate kinase: Function, regulation and role in cancer. Semin Cell Dev Biol (2015) 43:43–51. doi: 10.1016/j.semcdb.2015.08.004 26277545 PMC4662905

[51] Christofk HR Vander Heiden MG Harris MH Ramanathan A Gerszten RE Wei R. The M2 splice isoform of pyruvate kinase is important for cancer metabolism and tumour growth. Nature (2008) 452:230–3. doi: 10.1038/nature06734 18337823

[52] Christofk HR Vander Heiden MG Wu N Asara JM Cantley LC. Pyruvate kinase M2 is a phosphotyrosine-binding protein. Nature (2008) 452:181–6. doi:10.1038/nature06667 18337815

[53] Pirovich DB Da'dara AA Skelly PJ. Multifunctional fructose 1,6-bisphosphate aldolase as a therapeutic target. Front Mol Biosci (2021) 8:719678. doi: 10.3389/fmolb.2021.719678 34458323 PMC8385298

[54] Ahmed K Tunaru S Tang C Müller M Gille A Sassmann A. An autocrine lactate loop mediates insulin-dependent inhibition of lipolysis through GPR81. Cell Metab (2010) 11:311–9. doi: 10.1016/j.cmet.2010.02.012 20374963

[55] Kolko M Vosborg F Henriksen UL Hasan-Olive MM Diget EH Vohra R. Lactate transport and receptor actions in retina: potential roles in retinal function and disease. Neurochem Res (2016) 41:1229–36. doi: 10.1007/s11064-015-1792-x 26677077

[56] Sánchez-Chávez G Peña-Rangel MT Riesgo-Escovar JR Martínez-Martínez A Salceda R. Insulin stimulated-glucose transporter Glut 4 is expressed in the retina. PLoS One (2012) 7:e52959. doi: 10.1371/journal.pone.0052959 23285235 PMC3528717

[57] Al Hussein Al Awamlh S Wareham LK Risner ML Calkins DJ. Insulin signaling as a therapeutic target in glaucomatous neurodegeneration. Int J Mol Sci (2021) 22:4672. doi: 10.3390/ijms22094672 33925119 PMC8124776

[58] Fukasawa M Atsuzawa K Mizutani K Nakazawa A Usuda N. Immunohistochemical localization of mitochondrial fatty acid β-oxidation enzymes in rat testis. J Histochem Cytochem (2010) 58:195–206. doi: 10.1369/jhc.2009.954693 19875848 PMC2803708

[59] Zhang Y Deng WT Du W Zhu P Li J Xu F. Gene-based therapy in a mouse model of blue cone monochromacy. Sci Rep (2017) 7:6690. doi: 10.1038/s41598-017-06982-7 28751656 PMC5532293

[60] Popanda O Fox G Thielmann HW. Modulation of DNA polymerases alpha, delta and epsilon by lactate dehydrogenase and 3-phosphoglycerate kinase. Biochim Biophys Acta (1998) 1397:102–17. doi: 10.1016/S0167-4781(97)00229-7 9545551

[61] Castonguay Z Auger C Thomas SC Chahma M Appanna VD. Nuclear lactate dehydrogenase modulates histone modification in human hepatocytes. Biochem Biophys Res Commun (2014) 454:172–7. doi: 10.1016/j.bbrc.2014.10.071 25450376

[62] Huangyang P Simon MC. Hidden features: exploring the non-canonical functions of metabolic enzymes. Dis Model Mech (2018) 11:dmm033365. doi: 10.1242/dmm.033365 29991493 PMC6124551

[63] Zhang L Du J Justus S Hsu CW Bonet-Ponce L Wu WH. Reprogramming metabolism by targeting sirtuin 6 attenuates retinal degeneration. J Clin Invest (2016) 126:4659–73. doi: 10.1172/JCI86905 PMC5127684 27841758

[64] Takata N Miska JM Morgan MA Patel P Billingham LK Joshi N. Lactate-dependent transcriptional regulation controls mammalian eye morphogenesis. Nat Commun (2023) 14:4129. doi: 10.1038/s41467-023-39672-2 37452018 PMC10349100

[65] Townes-Anderson E. Increased levels of gene therapy may not be beneficial in retinal disease. Proc Natl Acad Sci USA (2013) 110:E1705. doi: 10.1073/pnas.1303746110 23553840 PMC3651456

[66] Zhang D Tang Z Huang H Zhou G Cui C Weng Y. Metabolic regulation of gene expression by histone lactylation. Nature (2019) 574:575–80. doi: 10.1038/s41586-019-1678-1 PMC6818755 31645732

[67] Liu C Wu J Zhu J Kuei C Yu J Shelton J. Lactate inhibits lipolysis in fat cells through activation of an orphan G-protein-coupled receptor, GPR81. J Biol Chem (2009) 284:2811–22. doi: 10.1074/jbc.M806409200 19047060

[68] Alfinito PD Townes-Anderson E. Activation of mislocalized opsin kills rod cells: a novel mechanism for rod cell death in retinal disease. Proc Natl Acad Sci USA (2002) 99:5655–60. doi: 10.1073/pnas.072557799 PMC122826 11943854

[69] Laroche S Stil A Germanin P Cherif H Chemtob H Bouchard J-F. Participation of L-lactate and its receptor HCAR1/GPR81 in neurovisual development. Cells (2021) 10:1640. doi: 10.3390/cells10071640 34208876 PMC8303161

[70] Schlatterer K Beck C Schoppmeier U Peschel A Kretschmer D. Acetate sensing by GPR43 alarms neutrophils and protects from severe sepsis. Commun Biol (2021) 4:928. doi: 10.1038/s42003-021-02427-0 34330996 PMC8324776

[71] Ito T Nakanishi Y Shibata R Sato N Jinnohara T Suzuki S. The propionate-GPR41 axis in infancy protects from subsequent bronchial asthma onset. Gut Microbes (2023) 15:2206507. doi: 10.1080/19490976.2023.2206507 37131293 PMC10158560

[72] An D Zeng Q Zhang P Ma Z Zhang H Liu Z. Alpha-ketoglutarate ameliorates pressure overload-induced chronic cardiac dysfunction in mice. Redox Biol (2021) 46:102088. doi: 10.1016/j.redox.2021.102088 34364218 PMC8353361

[73] Gupta R Wang M Ma Y Offermanns S Whim MD. The β-hydroxybutyrate-GPR109A receptor regulates fasting-induced plasticity in the mouse adrenal medulla. Endocrinology (2022) 163:bqac077. doi: 10.1210/endocr/bqac077 35595517 PMC9188660

[74] Li X Xie L Qu X Zhao B Fu W Wu B. GPR91, a critical signaling mechanism in modulating pathophysiologic processes in chronic illnesses. FASEB J (2020) 34:13091–105. doi: 10.1096/fj.202001037R 32812686

[75] Favret S Binet F Lapalme E Leboeuf D Carbadillo J Rubic T. Deficiency in the metabolite receptor SUCNR1 (GPR91) leads to outer retinal lesions. Aging (Albany NY) (2013) 5:427–44. doi: 10.18632/aging.100563 PMC3832265 23833031

[76] Chiang JYL Ferrell JM. Bile acid receptors FXR and TGR5 signaling in fatty liver diseases and therapy. Am J Physiol Gastrointest Liver Physiol (2020) 318:G554–g573. doi: 10.1152/ajpgi.00223.2019 31984784 PMC7099488

[77] Milligan G Alvarez-Curto E Hudson BD Prihandoko R Tobin AB. FFA4/GPR120: pharmacology and therapeutic opportunities. Trends Pharmacol Sci (2017) 38:809–21. doi: 10.1016/j.tips.2017.06.006 PMC5582618 28734639

[78] Joyal JS Sun Y Gantner ML Shao Z Evans LP Saba N. Retinal lipid and glucose metabolism dictates angiogenesis through the lipid sensor Ffar1. Nat Med (2016) 22:439–45. doi: 10.1038/nm.4059 PMC4823176 26974308

[79] Tan JK Mckenzie C Mariño E Macia L Mackay CR. Metabolite-sensing G protein-coupled receptors-facilitators of diet-related immune regulation. Annu Rev Immunol (2017) 35:371–402. doi: 10.1146/annurev-immunol-051116-052235 28446062

[80] Vohra R Aldana BI Bulli G Skytt DM Waagepetersen H Bergersen LH. Lactate-mediated protection of retinal ganglion cells. J Mol Biol (2019) 431:1878–88. doi: 10.1016/j.jmb.2019.03.005 30878479

[81] Vohra R Sanz-Morello B Tams ALM Mouhammad ZA Freude KK Hannibal J. Prevention of cell death by activation of hydroxycarboxylic acid receptor 1 (GPR81) in retinal explants. Cells (2022) 11:2098. doi: 10.3390/cells11132098 35805182 PMC9265426

[82] Nelson TS Simpson C Dyka F Dinculescu A Smith WC. A modified arrestin1 increases lactate production in the retina and slows retinal degeneration. Hum Gene Ther (2022) 33:695–707. doi: 10.1089/hum.2021.272 35081746 PMC9347377

[83] Zou GP Wang T Xiao JX Wang XY Jiang LP Tou FF. Lactate protects against oxidative stress-induced retinal degeneration by activating autophagy. Free Radic Biol Med (2023) 194:209–19. doi: 10.1016/j.freeradbiomed.2022.12.004 36493984

[84] Vohra R Kolko M. Lactate: more than merely a metabolic waste product in the inner retina. Mol Neurobiol (2020) 57:2021–37. doi: 10.1007/s12035-019-01863-8 31916030

[85] Xue Y Wang SK Rana P West ER Hong CM Feng H. AAV-Txnip prolongs cone survival and vision in mouse models of retinitis pigmentosa. Elife (2021) 10:e66240. doi: 10.7554/eLife.66240 33847261 PMC8081528

[86] Chen Y Zizmare L Trautwein C Paquet-Durand F. Measuring the release of lactate from wild-type and rd1 mouse retina. Adv Exp Med Biol (2023) 1415:429–34. doi: 10.1007/978-3-031-27681-1_63 37440068

[87] Do Rhee K Wang Y Ten Hoeve J Stiles L Nguyen TTT Zhang X. Ciliary neurotrophic factor-mediated neuroprotection involves enhanced glycolysis and anabolism in degenerating mouse retinas. Nat Commun (2022) 13:7037. doi: 10.1038/s41467-022-34443-x 36396639 PMC9672129

[88] Xu L Kong L Wang J Ash JD. Stimulation of AMPK prevents degeneration of photoreceptors and the retinal pigment epithelium. Proc Natl Acad Sci USA (2018) 115:10475–80. doi: 10.1073/pnas.1802724115 PMC6187182 30249643

[89] Fadden EJ Longley C Mahambrey T. Metformin-associated lactic acidosis. BMJ Case Rep (2021) 14:e239154. doi: 10.1136/bcr-2020-239154 PMC8268897 34244196

[90] Chang MY Tsai CY Chou LF Hsu SH Yang HY Hung CC. Metformin induces lactate accumulation and accelerates renal cyst progression in Pkd1-deficient mice. Hum Mol Genet (2022) 31:1560–73. doi: 10.1093/hmg/ddab340 34957500

[91] Aksu g. Aqueous humor lactic dehydrogenase activity in normal and diseased eyes. Ann Ophthalmol (1981) 13:1067–8. 7340658

[92] Salceda R Vilchis C Coffe V Hernández-Muñoz R. Changes in the redox state in the retina and brain during the onset of diabetes in rats. Neurochem Res (1998) 23:893–7. doi: 10.1023/A:1022467230259 9580389

[93] Simsek IB Artunay O. Evaluation of biochemical composition of vitreous of eyes of diabetic patients using proton magnetic resonance spectroscopy. Curr Eye Res (2017) 42:754–8. doi: 10.1080/02713683.2016.1242754 27897443

[94] Naik P Singh S Dave VP Ali MH Kumar A Joseph J. Vitreous D-lactate levels as a biomarker in the diagnosis of presumed infectious culture negative endophthalmitis. Curr Eye Res (2020) 45:184–9. doi: 10.1080/02713683.2019.1662057 31466487

[95] Samra YA Zaidi Y Rajpurohit P Raghavan R Cai L Kaddour-Djebbar I. Warburg effect as a novel mechanism for homocysteine-induced features of age-related macular degeneration. Int J Mol Sci (2023) 24:1071. doi: 10.3390/ijms24021071 36674587 PMC9865636

[96] Yokosako K Mimura T Funatsu H Noma H Goto M Kamei Y. Glycolysis in patients with age-related macular degeneration. Open Ophthalmol J (2014) 8:39–47. doi: 10.2174/1874364101408010039 25191529 PMC4150380

[97] Nolan ND Caruso SM Cui X Tsang SH. Renormalization of metabolic coupling treats age-related degenerative disorders: an oxidative RPE niche fuels the more glycolytic photoreceptors. Eye (Lond) (2022) 36:278–83. doi: 10.1038/s41433-021-01726-4 PMC8807833 34974542

[98] Kurihara T Westenskow PD Gantner ML Usui Y Schultz A Bravo S. Hypoxia-induced metabolic stress in retinal pigment epithelial cells is sufficient to induce photoreceptor degeneration. Elife (2016) 5:e14319. doi: 10.7554/eLife.14319 26978795 PMC4848091

[99] Morohoshi K Ohbayashi M Patel N Chong V Bird AC Ono SJ. Identification of anti-retinal antibodies in patients with age-related macular degeneration. Exp Mol Pathol (2012) 93:193–9. doi: 10.1016/j.yexmp.2012.03.007 22465421

[100] Yam M Engel AL Wang Y Zhu S Hauer A Zhang R. Proline mediates metabolic communication between retinal pigment epithelial cells and the retina. J Biol Chem (2019) 294:10278–89. doi: 10.1074/jbc.RA119.007983 PMC6664195 31110046

[101] Du J Zhu S Lim RR Chao JR. Proline metabolism and transport in retinal health and disease. Amino Acids (2021) 53:1789–806. doi: 10.1007/s00726-021-02981-1 PMC8054134 33871679

